# Predictors of success on the MCAT among post-baccalaureate pre-medicine students^☆^

**DOI:** 10.1016/j.heliyon.2020.e03778

**Published:** 2020-04-15

**Authors:** Rohini Ganjoo, Lisa Schwartz, Mackenzie Boss, Matthew McHarg, Yuliya Dobrydneva

**Affiliations:** a Department of Biomedical Laboratory Sciences, George Washington University School of Medicine and Health Sciences, Enterprise Hall, 44983 Knoll Square, Ashburn, Virginia, USA; b Department of Physician Assistant Sciences, George Washington University School of Medicine and Health Sciences, Enterprise Hall, 44983 Knoll Square, Ashburn, Virginia, USA

**Keywords:** Education, Admissions, Pre-medical, Post-baccalaureate, Advising

## Abstract

Post-baccalaureate pre-medicine programs (PBPMP) provide prerequisite coursework for non-life science majors who aspire to become physicians. Students entering these programs generally do not have previous college-level exposure to the natural sciences. This pilot study was conducted to determine characteristics of scientifically naive, career changer, pre-medical students that may be used by PBPMP admissions committees. Statistical analyses were performed between Medical College Admission Test (MCAT) scores and student gender, Scholastic Aptitude Test (SAT) scores, undergraduate field of study, and undergraduate Grade Point Average (GPA). While relationships between certain subscores on the SAT and MCAT were found, data suggest that other non-quantitative metrics be considered as predictors of performance among PBPMP students.

## 1 Introduction

Increasingly more non-traditional students are applying to medical school (allopathic and osteopathic programs), including those who did not consider themselves to be “pre-med” as undergraduates. Post-baccalaureate pre-medicine programs (PBPMP) offer unique opportunities to build a strong science foundation for career changing students who desire to become physicians. These programs are designed specifically to train individuals who were non-life science majors as undergraduate students and have not completed the life science courses required to apply to medical school, including general and organic chemistry, biology, physics and biochemistry. Alternatively, there are “record enhancer-type” of PBPMP that are typically offered at the graduate level, including certificate and master's degree programs, which allow students to augment their undergraduate GPA with graduate-level science coursework to make their application to medical schools more competitive (Andriole and Jeffe, 2011).

In the PBPMP described in the present paper, admitted students typically majored in social science or humanities as undergraduates. Admitted as a “cohort” in the summer semester, students complete 36 credit hours over a 12-month period, including 8 credits each of biology, physics, general chemistry, and organic chemistry, all with a laboratory component, as well as a 3-credit biochemistry course without laboratory. After successful completion of the PBPMP, graduates are prepared to take the standardized Medical College Admissions Test (MCAT) and apply to medical schools.

Admission to PBPMP is typically highly competitive, and admission to medical school upon completion of the program is not guaranteed. The matriculation rate to medical schools among program graduates is an important metric, which prospective students often consider when selecting a particular PBPMP program (Andriole and Jeffe, 2011). Therefore, programs strive to ensure a high success rate of admission to medical school among graduates, and in turn are motivated to admit only applicants who are predicted to have a high likelihood of success within the PBPMP and in the medical school admissions process. Successful medical school admission is dependent on a multitude of factors, including GPA, both undergraduate and graduate, with a focus on biology, chemistry, physics and math (BCPM) prerequisite coursework, performance on the MCAT, as well as competency in areas including interpersonal, intrapersonal, and thinking and reasoning (AAMC Core Competencies for Entering Medical Students, 2019).

However, there has been little previous research regarding what specific factors may predict success among applicants to a career changer-type PBPMP, since by the nature of such programs, applicants have completed little to no science coursework in their undergraduate programs. Thus, PBPMPs must consider other characteristics that may serve as predictors of success in their admissions processes. Since the medical school application process generally takes at least one-year post-completion of the PBPMP and is also dependent on numerous factors, performance on the MCAT exam was used as a proxy for estimating successful admission to medical school.

Predictably, previous research illustrates a positive correlation between both pre-admissions’ GPA and standardized test scores with performance in post-graduate health professions programs, such as medicine, nursing and athletic training. Mitchell (1990) conducted a systematic review of previous studies of traditional predictors of performance in medical school and noted that the undergraduate GPA most often included substantial coursework in biology, general and organic chemistry, physics, and math (BCPM), where non-science GPA was often judged as having less importance. Students in career changer-type PBPMP will have a greater proportion of their undergraduate GPA consisting of non-science-based coursework.

One study found that pre-admissions GPA had a 0.80 correlation to nursing school graduation GPA (Patzer et al., 2017), while another showed that high school GPA could predict 14% of the variance in student success in health professional programs (Platt et al., 2001). While these measurable variables serve as a way to objectively compare applicants and matriculants, there are a number of factors that often are not considered by admissions committees. For example, studies have found a relationship between age and medical school performance, noting that students over age 31 are at a higher risk for underperformance in their first year of medical school (Stratton and Elam, 2014).

Many of these studies define “success” in terms of medical school GPA or standardized test scores (e.g., USMLE), so we hypothesized that these same success indicators could be correlated to PBPMP student MCAT performance. High school graduates commonly take standardized exams, such as the SAT (Scholastic Achievement Test), that are considered in the college admissions process. Applicants who desire to become physicians are required to take the MCAT. The MCAT scores are reported as a Total, with median score of 500, and four subsections: Biological and Biochemical Foundations of Living Systems (Bio/Biochem), Chemical and Physical Foundations of Biological Systems (Chem/Phys), Psychological, Social, and Biological Foundations of Behavior (Psych/Soc), and Critical Analysis and Reasoning Skills (CARS; American Association of Medical Colleges, 2019). One would expect that previous academic performance could serve as a positive predictor of performance on the MCAT, yet career changer-type PBPMP applicants typically have undergraduate GPAs determined by little to no life science coursework. Therefore, performance on past standardized exams may serve as the best predictor of performance on the MCAT rather than undergraduate GPA. The objective of this study was to compare the relationship between SAT scores, undergraduate field of study, undergraduate GPA, and gender with student performance on the MCAT among a diverse group of pre-medical students enrolled in a career changer-type PBPMP.

## 2 Materials and methods

Retrospective data from the student academic records (N = 66, 4 cohorts) of the PBPMP were used to identify potential predictors of performance on the MCAT for students who have minimal prior academic exposure to the life sciences (“scientifically naive” students). Variables included undergraduate field of study, past standardized test scores (SAT), undergraduate GPA, and student gender (39 female and 27 male), which were correlated with Total MCAT score and individual MCAT sub-section scores. SAT scores were extracted from students' applications to the PBPMP for those who took the exam (N = 49). If more than one attempt on standardized testing was reported, only the first attempt scores on the SAT and MCAT were analyzed. Placement into undergraduate major or field of study groups was based upon the students’ self-reported major, as well as similarity of predominant coursework taken as an undergraduate student. Mean and standard error of the mean was calculated for each group, and t-tests or analysis of variance (ANOVA) were conducted to examine significant differences. Pearson correlation coefficient was calculated for each pair of quantitative variables.

## 3 Results

There was a significant difference [t (64) = -4.256, *p* = .009] between mean Total MCAT performance for males vs females. For females (N = 39; 59%), the mean Total MCAT score was 508.7 ± 1.0 and for males (N = 27; 41%) the average Total MCAT score was 513 ± 1.2. There was no correlation (-.048) between undergraduate GPA (N = 66) and mean MCAT Total score.

Each student (N = 66) was placed into one of seven undergraduate major categories as listed in Table 1. The mean GPA was calculated for each major category. There were significant differences in mean GPA across majors (F (6,54) = 2.284, *p* = .049), specifically between Applied Health Science vs all other majors, Health and Society vs Psychology, History, Arts and Humanities, Applied Health Sciences vs History, Arts, and Psychology, and Economics, Finance and Marketing vs History, Arts and Humanities, and Psychology. However, there was a moderate negative correlation (-.615, *p=*.142), which was not significant, between mean undergraduate GPA and mean MCAT Total for each major.

**Table 1:** Student undergraduate major and mean undergraduate GPA and MCAT Total score.

Undergraduate major	Students	Undergraduate GPA	Mean MCAT Total
Natural Sciences	7	3.2 ± 0.07	515 ± 2.9
Economics, Finance, Marketing	8	3.6 ± 0.06	513.8 ± 1.9
History and Political Science	10	3.5 ± 0.10	512.3 ± 2.1
Arts and Humanities	10	3.5 ± 0.07	511.2 ± 2.2
Psychology	14	3.5 ± 0.04	508.2 ± 1.3
Health and Society	6	3.6 ± 0.10	507.7 ± 2.6
Applied Health Sciences	6	3.6 ± 0.03	506.6 ± 1.9
Other	5	3.5 ± 0.10	508.2 ± 3.9

A one-way analysis of variance (ANOVA) was calculated to compare the mean MCAT Total score among undergraduate major categories. While there were no significant differences found among the major categories (*F* (6,54) = 2.071, *p* = .072), the mean MCAT Total score was highest at 515.0 for Natural Science (N = 7; 11%), which included students who had taken a significant number of physical science and math courses as undergraduates, including engineering majors, followed by 513.8 for Economics, Finance, and Marketing majors (N = 8; 12%), 512.3 for History and Political Science majors (N = 10; 15%), and 511.2 for Arts and Humanities majors (N = 10; 15%). Interestingly, those in the Applied Health Sciences (N = 6; 9%), including nursing, athletic training, and other majors for which grades for clinical rotations made up a large portion of the GPA, had the lowest mean MCAT Total score at 506.6. Students whose majors matched most closely to Health and Society (N = 6; 9%), including those in public health, had a mean MCAT Total of 507.7, and the largest group, Psychology majors (N = 14; 21%), had a 508.1 mean MCAT Total score (Table 1). A small number of students (N = 5; 8%) had undergraduate majors that did not fit into these major categories, and therefore their mean MCAT Total scores were not included in the ANOVA.

Next the relationship between two standardized examination metrics, the SAT and MCAT, was examined. Subscore analysis between the SAT and MCAT for students who took the SAT (N = 49; 74%) are displayed in Table 2. Forty-four (N = 44; 67%) students had an additional SAT Writing subscore as part of their SAT. The SAT Total score used (N = 49) included only the SAT Verbal and SAT Math subscores added together. For those who had the SAT Writing subscore, it was not included in the SAT Total calculation (N = 44). A moderate positive correlation was found between SAT Total and MCAT Total score (0.45) and MCAT CARS (0.52). Additionally, a moderate correlation was found between SAT Verbal and MCAT CARS (0.60), which is not unexpected given that both of these subscores focus on reading comprehension. Analysis of other metrics yielded only weak or no correlations between MCAT Total or subscores and SAT Total or Math, Verbal or Writing subscores of SAT (Table 2).

**Table 2:** Pearson correlation coefficient between standardized testing scores.

	SAT Total	SAT Verbal	SAT Math	SAT Writing (N = 44)
MCAT Total	0.45	0.39	0.38	0.43
MCAT Chem/Phys	0.31	0.22	0.31	0.32
MCAT CARS	0.52	0.60	0.28	0.33
MCAT Bio/BioChem	0.34	0.22	0.35	0.41
MCAT Psych/Soc	0.18	0.08	0.22	0.26

## 4 Discussion

This pilot study was the first to examine the relationship between certain demographic and academic metrics as predictors for MCAT performance among students with little previous life-science coursework enrolled in a career-changer type PBPMP at a mid-size, private, research university. While there was a significant relationship found between gender and mean MCAT Total scores, only moderate positive correlations were found between SAT Total and MCAT Total score (0.45) and MCAT CARS (0.52), and between the SAT Verbal and MCAT CARS subscores (0.60).

The students' undergraduate GPA was not correlated with the Total MCAT score (-.048) and may be due to the heterogeneity of student major, and thus diversity of coursework, often non-science in nature, contributing to the GPA. While males’ mean MCAT Total score was significantly higher than females, there was no relationship found between mean MCAT Total score and undergraduate major; both of these findings are consistent with a study by Gauer and Jackson (2018). However, it is not surprising to see that, on average, those students with some previous coursework in physical sciences (e.g., Natural Sciences) and math (e.g., Economics, Finance, and Marketing) tended to have the highest MCAT Total score. It is interesting to note that students who majored in health-related fields, such as Psychology, Health and Society, and the Applied Health Sciences, had the lowest MCAT Total scores on average. It is possible that these students initially aspired to a career in medicine but were discouraged by their performance in high school and early undergraduate science coursework.

In addition to the relatively small size of our sample (N = 66), it was challenging to categorize students into specific undergraduate fields of study because the title of the major and required coursework varied greatly across multiple undergraduate institutions. In addition, undergraduate GPA may be greatly impacted by the difference in academic rigor and grading policies across institutions. In reality students’ preparation for the SAT also may vary widely depending on whether they were being recruited for athletic teams and the quality of their high school coursework, which was not accounted for in the analyses.

Since the only correlations this study identified were between standardized test scores (SAT and MCAT), the findings suggest that other factors must be considered when reviewing applications for career-changer PBPMP. While a limitation of this study is its small sample size, and thus low statistical power to detect true but small differences, the results provide insight into admission metrics that could be indicative of future success.

In addition to quantitative factors, past studies (Cook et al., 2016; Pitt et al., 2012; Ray and Brown, 2015; Shaw and Coffman, 2017; Stratton and Elam, 2014; Todres et al., 2012) acknowledged a need to consider qualitative factors when considering an applicant for a medical, nursing, or physical therapy programs. Previous studies collected via interviews, written portions of a student's application, and personality tests have noted qualitative characteristics for admissions consideration that include grit (Ray and Brown, 2015), the ability to cope with difficulty (Pitt et al., 2012), ability to seek support (Todres et al., 2012), openness (Stratton and Elam, 2014), engagement with surroundings and peers (Todres et al., 2012), conscientiousness (Shaw and Coffman, 2017; Stratton and Elam, 2014), and self-reflection (Todres et al., 2012).

Burk-Rafel et al. (2019) found that undergraduate GPA and MCAT score were strong predictors of USMLE scores among medical students. However, the current pilot study did not follow PBPMP graduates long enough to analyze relationship with medical school performance or USMLE scores. These are metrics that may be considered for study in future research. In addition, recent literature on these topics is otherwise quite limited since significant changes were made to the MCAT in 2015.

For further study, more data will be accumulated through continuous enrollment into the PBPMP and longitudinal follow-up of graduates through medical school and beyond. Measures of other qualitative traits noted, including grit, persistence, and ethical decision-making, will also be introduced into our data collection and analyses. These qualitative assessments may provide insight into how an applicant may cope with both the academic and professional demands of a healthcare professional program beyond the metrics of standardized testing, particularly for career changer-type PBPMP applicants.

## 5 Conclusion

This pilot study contributes to the limited previous research regarding what specific factors may predict success among applicants to a career changer-type PBPMP who have completed little to no science coursework in their undergraduate programs. While relationships between certain subscores on the SAT and MCAT were found, similar relationships were not found between undergraduate GPA or major, suggesting that other non-quantitative metrics, including a more holistic approach, be considered as predictors of performance among PBPMP students.

## Declarations

### Author contribution statement

R. Ganjoo, Y. Dobrydneva, L. Schwartz: Conceived and designed the experiments; Performed the experiments; Analyzed and interpreted the data; Contributed reagents, materials, analysis tools or data; Wrote the paper.

M. McHarg, M. Boss: Contributed reagents, materials, analysis tools or data; Wrote the paper.

### Funding statement

This research did not receive any specific grant from funding agencies in the public, commercial, or not-for-profit sectors.

### Competing interest statement

The authors declare no conflict of interest.

### Additional information

No additional information is available for this paper.
